# A Childhood Farm Environment Protects from Allergic Sensitization until Middle Age but Not from New-Onset Sensitization in Adulthood: A 15 Year Longitudinal Study

**DOI:** 10.3390/ijerph18137078

**Published:** 2021-07-02

**Authors:** Anna Karoliina Haarala, Suvi-Päivikki Sinikumpu, Eeva Vaaramo, Jari Jokelainen, Markku Timonen, Juha Auvinen, Juha Pekkanen, Laura Huilaja

**Affiliations:** 1 Department of Dermatology, University Hospital of Oulu, Finland and Medical Research Center, PEDEGO Research Group, University of Oulu, 90014 Oulu, Finland; karoliina.hulkko@student.oulu.fi (A.K.H.); suvi-paivikki.sinikumpu@oulu.fi (S.-P.S.); 2 Infrastructure for Population Studies, Faculty of Medicine, University of Oulu, 90014 Oulu, Finland; eeva.vaaramo@oulu.fi (E.V.); jari.jokelainen@oulu.fi (J.J.); 3 Center for Life Course Health Research, Faculty of Medicine, University of Oulu, 90014 Oulu, Finland; markku.timonen@oulu.fi (M.T.); juha.auvinen@oulu.fi (J.A.); 4 Medical Research Center, Oulu University Hospital, 90220 Oulu, Finland; 5 Department of Public Health, University of Helsinki, 00014 Helsinki, Finland; juha.pekkanen@helsinki.fi; 6 Department of Health Security, Environmental Health, Finnish Institute for Health and Welfare, 00271 Kuopio, Finland

**Keywords:** adulthood, childhood, farm, new onset, sensitization

## Abstract

Data are insufficient on the protective effect of a farm environment in childhood regarding sensitization in middle age and new-onset sensitization in adulthood. A skin prick test (SPT) and questionnaire data from the Northern Finland Birth Cohort 1966 study (NFBC66) were used to investigate sensitization at age 46 years related to childhood living environment. A subpopulation of 3409 participants was analyzed to study factors related to new-onset sensitization between ages of 31 and 46 years. Data on complete SPTs were available for 5373 cohort members at age 46. Professional farming by parents (odds ratio (OR) 0.54; 95% confidence interval (CI) 0.43–0.68) and keeping of farm animals (OR 0.53; 95% CI 0.43–0.66) in infancy were associated with a lower risk of sensitization at age 46. Sensitization (OR 0.58; 95% CI 0.47–0.72) and polysensitization (OR 0.43; 95% CI 0.32–0.57) were less common in those who lived in a rural area in infancy compared to a city area. The childhood living environment had no effect on new-onset sensitization between ages 31 and 46. We conclude that living on a farm or in a rural environment in childhood had a protective effect on sensitization even in middle age, but these factors did not protect from new-onset sensitization in adults.

## 1. Introduction

Allergic diseases are a major health challenge worldwide [1] and the prevalence of atopic sensitization in adults has been reported as still increasing in industrialized countries such as those in Northern Europe in the current millennium [2,3]. Allergic diseases have a complex background where genetics, environmental factors, and timing play a role. Factors early in life and during the fetal period are thought to be important influences on the risk of sensitization later in life [4,5].

Although the farm effect during childhood, currently considered an indicator for exposure to a diversity of microbes [6,7,8], is an important protective factor for future aeroallergen sensitization in several studies [9,10,11,12], most of these only reach until early adulthood. In a cross-sectional study on sensitization and asthma conducted in Finland, a childhood farm environment had a protective effect on sensitization to pollens and cat in subjects aged 18–26 years [9]. In another Finnish study of unselected adults, a farming environment in childhood was found to protect from sensitization at the age of 31 [10]. In Denmark, the effect of a childhood living environment on sensitization was studied in 1236 men aged 30 to 40 years. This study found not only the overall prevalence of sensitization, but also specific sensitization decreased with a decreasing degree of urbanization, being lowest in the farm group [11]. The effect of a current living environment in adulthood versus sensitization was investigated [13]. In a study conducted in Finland, women who lived on a farm were less likely to have sensitization to pollens and cat than those who did not. This association was strongest in those who had also lived on a farm in childhood [13].

Currently, there are limited data on the effect of early farm exposure on sensitization to aeroallergens later in adulthood. Previous studies have not analyzed these effects with an extensive prospective follow-up, neither have studies analyzed the effect of a childhood environment on new-onset sensitization in adulthood. The aims of the present study were to examine the effect of childhood farm exposure on the risk of sensitization and polysensitization at age 46 years and the effect of a childhood living environment on new-onset sensitization between the ages of 31 and 46.

## 2. Methods

### 2.1. Study Population

**Figure 1:**
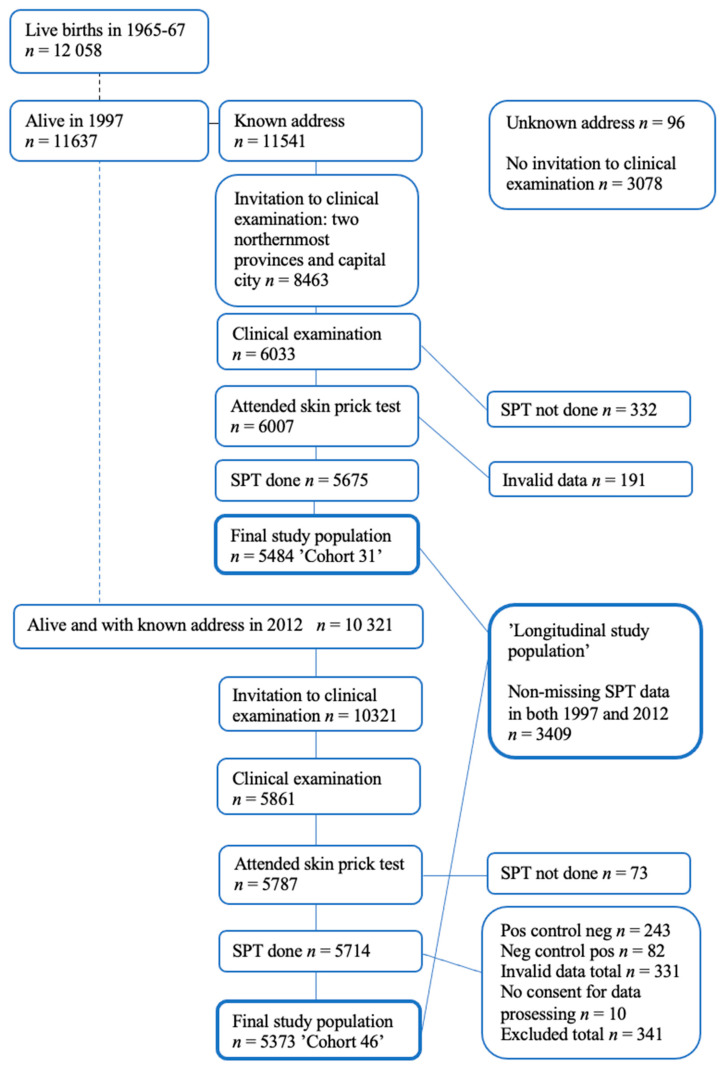
Flow chart of the NFBC 1966 population: follow-up visits including skin prick tests (SPTs).

Our study data originated from the Northern Finland Birth Cohort 1966 (NFBC66), a longitudinal research program in the two northernmost provinces in Finland. The NFBC66 initially included all 12,058 children whose expected date of birth fell in the year 1966. The children of NFBC66 were followed regularly since their birth. The follow-up was conducted through health questionnaires and clinical investigations including skin examination [14,15]. To date, the cohort has been subjected to four main follow-up visits: at birth, and at 14, 31, and 46 years. Skin prick tests (SPTs) were performed at the ages of 31 and 46 years (Figure 1) [3]. Detailed information on the NFBC66 can be found on the research program’s website [16].

### 2.2. Skin Prick Test

SPTs were conducted with standard dilutions of three of the most common allergens in Finland (cat, birch, and timothy grass), plus house dust mite (HDM) (*Dermatophagoides Pteronyssinus*) (Alk-Abello Nordic, Espoo, Finland) as described previously [3]. 

### 2.3. Questionnaire and Confounder Factors

Data on environmental factors in childhood were collected with health questionnaires antenatally and at age 31. The questionnaires included information on the place of residence in infancy, the parents’ professional farming and keeping of farm animals, keeping of cats and dogs, and the number of animal species. Potential confounders were selected on the basis of previous studies that showed an association with sensitization: residential density [10], maternal smoking during pregnancy [17], maternal and paternal asthma [18,19], maternal and paternal allergy [18,19], mother’s age of menarche [20], maternal body mass index (BMI) [21], parity [18], maternal age [22], and study subject’s gestational age [18], sex [23,24], current socio-economic status (SES, defined as level of education) [25], current BMI [26], current residence on a farm [12,27] and current smoking [28] (defined as smoking at least once a week) were included. Maternal education and study subject’s birth weight and height were considered but ultimately excluded from the final analyses due to the absence of a significant association with sensitization. Analyses included gestational age, the alternative confounder for birth height and weight.

### 2.4. Statistical Analysis

Associations between the environmental factors in infancy and sensitization and polysensitization at age 46 years were analyzed by cross-tabulation and the data are presented as frequencies and percentages. The chi-squared test was used to test differences between sensitized and non-sensitized and classes of polysensitization. Associations between environmental factors in infancy and sensitization or polysensitization at age 46 years were tested using binary logistic regression analysis and multinomial logistic regression. Two models were used, unadjusted (crude) and adjusted by sex, maternal age, smoking during pregnancy, maternal BMI, residential density, current education, current BMI, current farm living, current smoking, paternal asthma, paternal allergy, maternal asthma, maternal allergy, gestational age, mother’s age of menarche and parity. Analyses were conducted using the SAS software package (version 9.4, SAS Institute Inc., Cary, NC, USA).

## 3. Results

### 3.1. Characteristics of the Study Population

At 46 years, invitations were sent to every living member of the cohort whose addresses were known (*n* = 10,321) and, of these, 5861 participants attended the clinical examination day. In the 46-year follow-up study, SPTs were performed on 5714 (55.4% of invited) participants. Because of invalid data, 331 participants were excluded from the 46 -year follow-up analysis and a further 10 participants did not provide their consent to data processing. The final study population consisted of participants who had complete SPT data on all allergens at age 46 (*n* = 5373) of which 2394 (44.6%) were men and 2979 (55.4%) were women. A subpopulation of 3409 participants with complete data on all allergens at age 31 and 46 years (longitudinal subpopulation) was further analyzed (Figure 1).

From the final study population (*n* = 5373), professional farming by parents and keeping of farm animals were reported by 1370 (25.5%) and 1526 (28.4%) participants, respectively. Place of residence was divided into three categories: city (*n* = 1757/32.7%), village (*n* = 1394/25.9%), and outlying district (*n* = 2203/41.0%). Approximately one-third of the participants (*n* = 1728/32.2%) had cats and dogs (*n* = 1918/35.7%) as pets under age 7 years.

### 3.2. Environmental Factors and Allergic Sensitization at Age 46

After adjusting for multiple potential confounders, the risk of allergic sensitization remained significantly lower in those who lived in outlying districts compared to cities (odds ratio (OR) 0.58; 95% confidence interval (CI) 0.47–0.72), and in those whose parents were professional farmers (OR 0.54; 95% CI 0.43–0.68) or had farm animals (OR 0.53; 95% CI 0.43–0.66). The protective effect of cats (OR 0.74; 95% CI 0.62–0.89) and dogs (OR 0.74; 95% CI 0.62–0.88) remained significant after adjustment. The risk of sensitization was significantly and inversely associated with the number of animal species with an OR of 0.41 (95% CI 0.31–0.54) for three or more animal species (no farm animals as a reference). See Table 1.

The risk of polysensitization was significantly lower in those who lived in an outlying district compared to a city (OR 0.43; CI 0.32–0.57). Professional farming by parents (OR 0.34; 95% CI 0.24–0.49) and keeping of farm animals (OR 0.34; 95% CI 0.25–0.48) were both associated with a significantly lower risk for polysensitization. An increasing number of farm animals gradually lowered the risk of polysensitization (OR 0.26; 95% CI 0.17–0.40 for three or more animal species (no farm animals as a reference)). See Table 2.

When analyzed by allergen, the keeping of three or more animal species on the farm had the strongest protective effect on sensitization to timothy grass (OR 0.27; 95% CI 0.18–0.40), followed by cat (OR 0.34; 95% CI 0.23–0.50), and birch (OR 0.48; 95% CI 0.34–0.68), but had no effect on sensitization to HDM (OR 0.58; 95% CI 0.32–1.04). Residence in an outlying district protected from sensitization in the same pattern. Parents’ professional farming and keeping of farm animals decreased the risk of sensitization to pollen and cat, but not to HDM. Having at least one dog in the family before the participant turned 7 years was associated with a lower risk of sensitization to HDM (OR 0.63; 95% CI 0.43–0.91) (Table 3).

### 3.3. Environmental Factors and New-Onset Sensitization between Ages 31 and 46 Years

From the longitudinal subpopulation (*n* = 3409), two subgroups were further analyzed: the first group stayed unsensitized (no positive SPT findings) during the follow-up period from age 31 to 46 years, while the second group acquired sensitization (no positive SPT at 31 and ≥1 positive SPT at 46 years) during this period. Neither farm exposure nor place of residence in infancy differed between these two groups (Table 4). Maternal asthma was the only statistically significant confounder and was more common in those who acquired sensitization during this period (data not shown).

## 4. Discussion

From this large birth cohort study, we showed that living on a farm and especially the keeping of farm animals in childhood had a protective effect on sensitization and polysensitization to aeroallergens even in middle-aged adults. This is in line with the hygiene hypothesis, highlighting the importance of environmental diversity [29]. Living in an outlying district in childhood was associated with a decreased risk of sensitization compared to living in a city. The keeping of cats and dogs had a protective effect on sensitization and the risk of sensitization decreased with an increasing number of animal species. However, these childhood factors did not protect from new-onset sensitization acquired between the ages of 31 and 46 years.

In previous studies, the effect of a childhood farm environment on aeroallergen sensitization was commonly protective and our present findings confirm this perception. Although a birth cohort study conducted in Finland found that the overall sensitization rate to common aeroallergens at age 31 was lower in those with a farm background in childhood (*n* = 5509), the specific aeroallergens were not examined separately in that study [10]. A Danish study found a lower risk of both overall and specific sensitization in the childhood animal farm group among male subjects aged 30 to 40 years (*n* = 1236) compared to rural, town, or city habitants. However, the study population was smaller than our study and excluded women. Moreover, the median age of the population was less than 35 years, and thus unrepresentative of a middle-aged population [11].

Studies have reported differing results when the effect of farming was examined by allergen. Although in the previously represented Danish study, sensitization to birch, grass, cat, and HDM was less common in participants with a farm background [11], others reported differing findings. For instance, in a Finnish study of 18–26-year-old subjects (*n* = 296), a childhood farming environment led to less sensitization to pollens and cat in the SPT [9]. However, significantly higher sensitization rate to HDM was found in those with a farming background [9]. In our study, sensitization to birch, timothy grass, and cat allergens was less common if the parents were professional farmers or if the family had farm animals. Still, these factors did not protect from sensitization to HDM.

In the present study, the same protective associations were also observed for polysensitization, which was linked to an increased risk of allergic multimorbidity [30,31,32]. Atopic dermatitis (AD) is known to increase the risk of atopic comorbidities (allergic rhino-conjunctivitis, allergic asthma, and IgE-mediated food allergy), and this atopic march may start developing in the early years of life [33,34]. In addition to the protective effect on sensitization, studies also showed the protective effect of early farm exposure on asthma [9] and allergic rhinitis [35,36], thus also highlighting the clinical relevance of the lower polysensitization rate found in the farm group. 

Differences in sensitization between sexes have been proposed previously, with men seemingly more prone to sensitization [3,23,24]. This was also found in our results as the male sex was a risk factor for overall sensitization, specific sensitization, and polysensitization.

Sensitization to common aeroallergens is less common in adult farmers. A Finnish study of 433 women, including 231 women currently living on a farm, found a reduced risk of sensitization to pollens and cat in the farm group. In addition, the protective effect was most pronounced in those with both childhood and current farming exposure [13]. In the present study, even when adjusted for current farming, the protective effect of childhood farm exposure remained significant.

The other remarkable finding in our study is that a farm environment in childhood did not protect from new-onset sensitization in adults. In longitudinal analyses (between ages 31 and 46 years), there was no difference in the effect of childhood environment (living on a farm or rural environment and having a pet) between new-onset and non-sensitized groups. In our study, only the mother’s asthma was associated with a higher risk of sensitization during the follow-up period from 31 to 46 years of age. Although new-onset sensitization can occur in adults [3,37] most sensitization likely occurs early in life. This suggests that some other factor independent of a childhood environment drives the process in those who develop sensitization later in life.

This is the first large scale study on the effect of childhood living environment on sensitization that reaches until the age of 46 years. The strengths of the study include its unselected population and the longitudinal follow-up data, which enabled the analysis of childhood environmental factors for sensitization in middle age. The prospective setting of the study excludes recall bias. Only data on maternal and paternal asthma, maternal and paternal allergy, and pet keeping under the age of 7 years were recorded retrospectively. Potential confounders were included in the analysis comprehensively. The relatively high participation rate allows our results to be generalized to the entire population. We used the SPT technique, considered the gold standard for detecting allergic sensitization [38] with its good positive predictive value, to determine clinical allergy in respiratory allergic diseases [39]. In previous studies, the effect of farming was not always subdivided concerning farm animals, the variety of animal species, and pets. We were able to break down the effect of farming and showed that farm animals and pets had a protective association with sensitization and that the number of animal species affected the risk of sensitization. After adjusting for current living on a farm, we showed that the protective association between childhood exposure to a farm environment and sensitization remained significant. 

The lack of a concurrent specific immunoglobulin E assessment can be considered a limitation of this study. The duration of farm exposure in childhood was not recorded and we cannot definitively state that the antenatally reported environment stayed the same after birth. Some participants declined the SPT for unknown reasons. Consequently, some of the most sensitized individuals may not have been included in the analyzed population. Furthermore, there was a possibility of selection bias in the population who participated in the clinical examinations. The permitted use of antihistamines may also have affected the outcome of the SPT, although few among the study population reported current antihistamine medication.

## 5. Conclusions

In conclusion, environmental exposures early in life are important in developing tolerance to aeroallergens and this protective effect is shown to last until middle age. Therefore, measures aiming to increase contact with natural environments and their diversity of microbes are essential in childhood.

## Figures and Tables

**Table 1:** Adjusted associations between environmental factors in infancy and sensitization to any of the four aeroallergens at age 46.

			Sensitization at Age 46	
Environmental Factors in Infancy		N ^a^ (%)	*n* (%)	Adjusted ^b^ OR (95 % CI)
Place of residence	City	1757 (32.8)	651 (37.1)	Reference
	Village	1394 (26.0)	448 (32.1)	0.89 (0.72–1.11)
	Outlying districts	2203 (41.2)	543 (24.7)	0.58 (0.47–0.72)
Professional farming	No	3758 (73.3)	1258 (33.5)	Reference
	Yes	1370 (26.7)	296 (21.6)	0.54 (0.43–0.68)
Farm animals	No	3788 (71.3)	1292 (34.1)	Reference
	Yes	1526 (28.7)	339 (22.2)	0.53 (0.43–0.66)
Keeping of cats ^c^	No	1853 (51.8)	639 (34.5)	Reference
	Yes	1728 (48.3)	446 (25.8)	0.74 (0.62–0.89)
Keeping of dogs ^c^	No	1690 (46.8)	586 (34.7)	Reference
	Yes	1918 (53.2)	502 (26.2)	0.74 (0.62–0.88)
Animal species	0	1006 (28.4)	379 (37.7)	Reference
	1	1010 (28.5)	350 (34.7)	0.91 (0.74–1.13)
	2	691 (19.5)	171 (24.8)	0.63 (0.48–0.81)
	≥3	835 (23.6)	153 (19.6)	0.41 (0.31–0.54)

^a^ Total number of participants varies due to incomplete response to questionnaire, ^b^ adjusted for sex, maternal age, smoking during pregnancy, maternal BMI, residential density, current education, current BMI, current farm living, current smoking, paternal asthma, paternal allergy, maternal asthma, maternal allergy, gestational age, mother’s age of menarche and parity, ^c^ before age 7 years.

**Table 2:** Adjusted associations between environmental factors in infancy and monosensitization or polysensitization at age 46.

		Monosensitization at Age 46		Polysensitization ^a^ at Age 46	
Environmental Factors in Infancy	N ^b^ (%)	*n* (%)	Adjusted ^c^ OR (95 % CI)	*n* (%)	Adjusted ^c^ OR (95 % CI)
Place of residence					
City	1757 (32.8)	307 (17.5)	Reference	344 (19.6)	Reference
Village	1394 (26.0)	213 (15.3)	0.86 (0.65–1.13)	235 (16.9)	0.93 (0.71–1.22)
Outlying districts	2203 (41.2)	323 (14.7)	0.74 (0.57–0.95)	220 (10.0)	0.43 (0.32–0.57)
Professional farming					
No	3758 (73.3)	615 (16.4)	Reference	643 (17.1)	Reference
Yes	1370 (26.7)	190 (13.9)	0.72 (0.56–0.94)	106 (7.7)	0.34 (0.24–0.49)
Farm animals					
No	3788 (71.3)	623 (16.5)	Reference	669 (17.7)	Reference
Yes	1526 (28.7)	213 (14.0)	0.71 (0.55–0.92)	126 (8.3)	0.34 (0.25–0.48)
Keeping of cats ^d^					
No	1853 (51.8)	299 (16.1)	Reference	340 (18.4)	Reference
Yes	1728 (48.3)	271 (15.7)	0.95 (0.76–1.19)	175 (10.1)	0.55 (0.43–0.70)
Keeping of dogs ^d^					
No	1690 (46.8)	285 16.9)	Reference	301 (17.8)	Reference
Yes	1918 (53.2)	287 (15.0)	0.86 (0.69–1.06)	215 (11.2)	0.62 (0.49–0.79)
Animal species					
0	1006 (28.4)	160 (15.9)	Reference	219 (21.8)	Reference
1	1010 (28.5)	183 (18.1)	1.13 (0.85–1.49)	167 (16.5)	0.76 (0.58–1.00)
2	691 (19.5)	112 (16.2)	0.93 (0.67–1.28)	59 (8.5)	0.40 (0.27–0.57)
≥ 3	835 (23.6)	105 (12.6)	0.59 (0.42–0.84)	59 (7.1)	0.26 (0.17–0.40)

^a^ ≥2 positive skin prick tests, ^b^ total number of participants varies due to incomplete responses to the questionnaire, ^c^ multinomial logistic regression. Adjusted for sex, maternal age, smoking during pregnancy, maternal BMI, residential density, current education, current BMI, current farm living, current smoking, paternal asthma, paternal allergy, maternal asthma, maternal allergy, gestational age, mother’s age of menarche and parity, ^d^ before age of 7 years.

**Table 3:** Adjusted associations between environmental factors in infancy and sensitization to a specific aeroallergen at age 46 years.

		Sensitization to Cat at Age 46		Sensitization to Birch at Age 46		Sensitization to Timothy Grass at Age 46		Sensitization to HDM at Age 46	
Environmental Factors in Infancy	N ^a^ (%)	*n* (%)	Adjusted ^b^ OR (95 % CI)	*n* (%)	Adjusted ^b^ OR (95 % CI)	*n* (%)	Adjusted ^b^ OR (95 % CI)	*n* (%)	Adjusted ^b^ OR (95 % CI)
Place of residence									
City	1757 (32.8)	343 (19.5)	Reference	359 (20.4)	Reference	379 (21.6)	Reference	91 (5.2)	Reference
Village	1394 (26.0)	225 (16.1)	0.90 (0.69–1.16)	254 (18.2)	1.00 (0.78–1.29)	236 (16.9)	0.80 (0.61–1.04)	68 (4.9)	0.87 (0.55–1.38)
Outlying districts	2203 (41.2)	257 (11.7)	0.52 (0.40–0.67)	289 (13.1)	0.66 (0.51–0.86)	235 (10.7)	0.47 (0.36–0.61)	97 (4.4)	0.79 (0.51–1.22)
Professional farming									
No	3758 (73.3)	655 (17.4)	Reference	700 (18.6)	Reference	680 (18.1)	Reference	189 (5.0)	Reference
Yes	1370 (26.7)	125 (9.1)	0.42 (0.30–0.57)	151 (11.0)	0.60 (0.45–0.79)	118 (8.6)	0.35 (0.25–0.50)	56 (4.1)	0.98 (0.63–1.53)
Farm animals									
No	3788 (71.3)	672 (17.7)	Reference	722 (19.1)	Reference	710 (18.7)	Reference	192 (5.1)	Reference
Yes	1526 (28.7)	148 (9.7)	0.41 (0.31–0.56)	175 (11.5)	0.58 (0.44–0.76)	133 (8.7)	0.40 (0.29–0.55)	63 (4.1)	0.87 (0.56–1.35)
Keeping of cats ^c^									
No	1853 (51.8)	342 (18.5)	Reference	355 (19.2)	Reference	357 (19.3)	Reference	84 (4.5)	Reference
Yes	1728 (48.3)	210 (12.2)	0.70 (0.56–0.88)	227 (13.1)	0.76 (0.61–0.94)	185 (10.7)	0.52 (0.41–0.67)	85 (4.9)	1.00 (0.69–1.46)
Keeping of dogs ^c^									
No	1690 (46.8)	314 (18.6)	Reference	331 (19.6)	Reference	311 (18.4)	Reference	94 (5.6)	Reference
Yes	1918 (53.2)	235 (12.3)	0.68 (0.54–0.84)	256 (13.4)	0.71 (0.57–0.88)	231 (12.0)	0.67 (0.53–0.84)	75 (3.9)	0.63 (0.43–0.91)
Animal species									
0	1006 (28.4)	210 (20.9)	Reference	226 (22.5)	Reference	223 (22.2)	Reference	54 (5.4)	Reference
1	1010 (28.5)	185 (18.3)	0.92 (0.71–1.19)	181 (17.9)	0.82 (0.63–1.06)	177 (17.5)	0.75 (0.57–0.98)	47 (4.7)	0.71 (0.44–1.15)
2	691 (19.5)	72 (10.4)	0.50 (0.36–0.71)	84 (12.2)	0.56 (0.40–0.77)	68 (9.8)	0.42 (0.30–0.60)	33 (4.8)	0.90 (0.54–1.50)
≥3	835 (23.6)	68 (8.1)	0.34 (0.23–0.50)	83 (9.9)	0.48 (0.34–0.68)	64 (7.7)	0.27 (0.18–0.40)	33 (4.0)	0.58 (0.32–1.04)

^a^ Total number of participants varies due to incomplete responses to the questionnaire; ^b^ Adjusted for sex, maternal age, smoking during pregnancy, maternal BMI, residential density, current education, current BMI, current farm living, current smoking, paternal asthma, paternal allergy, maternal asthma, maternal allergy, gestational age, mother’s age of menarche and parity; ^c^ Before age 7; HDM: house dust mite.

**Table 4:** The risk of acquired sensitization between ages 31 and 46 years related to environmental factors in infancy (longitudinal subpopulation).

			Acquired Sensitization from Age 31 to 46	
Environmental Factors in Infancy		N ^a^ (%)	*n* (%)	Adjusted ^b^ OR (95 % CI)
Place of residence	City	703(29.0)	79 (11.2)	Reference
	Village	586 (24.2)	57 (9.7)	0.91(0.60–1.37)
	Outlying districts	1137 (46.9)	107 (9.4)	0.84 (0.58–1.23)
Professional farming	No	1586 (68.0)	167 (10.5)	Reference
	Yes	745 (32.0)	65 (8.7)	0.84(0.58–1.22)
Farm animals	No	1585 (65.8)	172 (10.9)	Reference
	Yes	823 (34.2)	71(8.6)	0.75 (0.52, 1.09)
Keeping of cats ^c^	No	1111 (47.7)	113 (10.2)	Reference
	Yes	1217 (52.3)	123 (10.1)	1.09 (0.79–1.51)
Keeping of dogs ^c^	No	1020 (43.4)	113 (11.1)	Reference
	Yes	1332 (56.6)	125 (9.4)	0.79 (0.58–1.09)
Animal species	0	574 (25.2)	65 (11.2)	Reference
	1	618 (27.2)	67 (10.8)	0.88(0.58–1.34)
	2	490 (21.5)	52 (10.6)	1.05(0.67–1.64)
	≥3	594 (26.1)	49 (8.3)	0.64(0.39–1.04)

^a^ Total number of participants varies due to incomplete responses to the questionnaire, ^b^ adjusted for sex, maternal age, smoking during pregnancy, maternal BMI, residential density, current education, current BMI, current farm living, current smoking, paternal asthma, paternal allergy, maternal asthma, maternal allergy, gestational age, mother’s age of menarche and parity, ^c^ before age 7 years.

## Data Availability

The data that support the findings of this study are available from Northern Finland Birth Cohort 1966 Study. Restrictions apply to the availability of these data, which were used under license for this study. Data are available at http://www.oulu.fi/nfbc/node/44315 with the permission of Northern Finland Birth Cohort (accessed on 20 May 2021).
